# Run-and-Tumble Dynamics and Mechanotaxis Discovered in Microglial Migration

**DOI:** 10.34133/research.0063

**Published:** 2023-03-10

**Authors:** Yiyu Zhang, Da Wei, Xiaochen Wang, Boyi Wang, Ming Li, Haiping Fang, Yi Peng, Qihui Fan, Fangfu Ye

**Affiliations:** ^1^ Beijing National Laboratory for Condensed Matter Physics, Institute of Physics, Chinese Academy of Sciences, Beijing 100190, China.; ^2^ School of Physical Sciences, University of Chinese Academy of Sciences, Beijing 100049, China.; ^3^ Wenzhou Institute, University of Chinese Academy of Sciences, Wenzhou, Zhejiang 325000, China.; ^4^ Songshan Lake Materials Laboratory, Dongguan, Guangdong 523808, China.; ^5^ School of Science, East China University of Science and Technology, Shanghai 200237, China.; ^6^ Oujiang Laboratory (Zhejiang Lab for Regenerative Medicine, Vision and Brain Health), Wenzhou, Zhejiang 325000, China.

## Abstract

Microglia are resident macrophage cells in the central nervous system that search for pathogens or abnormal neural activities and migrate to resolve the issues. The effective search and targeted motion of macrophages mean dearly to maintaining a healthy brain, yet little is known about their migration dynamics. In this work, we study microglial motion with and without the presence of external mechanostimuli. We discover that the cells are promptly attracted by the applied forces (i.e., mechanotaxis), which is a tactic behavior as yet unconfirmed in microglia. Meanwhile, in both the explorative and the targeted migration, microglia display dynamics that is strikingly analogous to bacterial run-and-tumble motion. A closer examination reveals that microglial run-and-tumble is more sophisticated, e.g., they display a short-term memory when tumbling and rely on active steering during runs to achieve mechanotaxis, probably via the responses of mechanosensitive ion channels. These differences reflect the sharp contrast between microglia and bacteria cells (eukaryotes vs. prokaryotes) and their environments (compact tissue vs. fluid). Further analyses suggest that the reported migration dynamics has an optimal search efficiency and is shared among a subset of immune cells (human monocyte and macrophage). This work reveals a fruitful analogy between the locomotion of 2 remote systems and provides a framework for studying immune cells exploring complex environments.

## Introduction

Motility takes different forms for life across scales [1]. Swimming by flagella and cilia and migration by pseudopodia are the primary forms of microbial motility [2]. With these motilities, microorganisms explore their environment and fulfill various functions such as feeding, predating, and mating [2,3]. In the meantime, diverse patterns of locomotion arise from such explorations. Some patterns can be simply decomposed to consecutive straight movements (“run”) and turns, which are attributed to 2 or 3 distinct motility states. Examples include the run-and-tumble motion of peritrichous bacteria (e.g., *E. coli*) [4], the run-reverse-flick motion of most marine bacteria which are monotrichous (e.g., *P. citronellolis* and *V. alginolyticus*) [5,6], and the tristate polygonal pattern displayed by uniflagellated protist *E. gracilis* [7]. However, the patterns easily get much more complex. For example, the motion of protists with multiple flagella/cilia (e.g., *T. subcordiformis* and *Paramecium*) often comprises numerous gaits that cannot be easily abstracted [8,9].

Among these motions, the best-understood case, and arguably the simplest one, is the run-and-tumble pattern that consists of smooth swimming (“run”) interrupted by abrupt turnings (“tumble”). It is firstly observed in the chemotactic motion of *E. coli* in fluidic environment [4]. Interestingly, bistate motility also exists in some migratory cells exploring more complex environments (e.g., in tissues) [10,11,12,13]. Compared to the well-established study of microbial swimming, this is still a burgeoning field and much of the cells’ migration dynamics remains to be explored.

Immune cells mark an important category of migratory cells. Microglia, the primary immune cells in the brain [14], serve both immunological functions (e.g., approaching and removing the pathogens) and developmental functions (e.g., synapse pruning). They have long been considered quiescent until recent efforts reveal respectively the rapid extension of their branches [15] and their whole-cell migration in chemical gradients [16]. In addition to chemical cues, mechanical stimuli are gradually considered as another potentially important mechanism to guide cell migration [17,18]. Exploring their targeted motion toward external cues (tactic behaviors) and the associated motility patterns will advance our knowledge of this crucial immune cell [19].

In this study, we examine how microglia respond to mechanostimuli and characterize their migration dynamics. Our results reveal the mechanotaxis of microglia, which is previously unknown in this cell. Moreover, we find the microglial migration dynamics to be strikingly analogous to the run-and-tumble motion of bacteria—despite that drastic differences exist between the 2 cells, their environments, and their goal of motion. Closer examination also sheds light on the peculiarities of microglial motility. For example, microglia display a memory effect in their tumbles and rely on active steering during runs to approach the target via the responses of mechanosensitive ion channels. We also explore other macrophage-like cells for similar migration dynamics. Preliminary results suggest that such dynamics is shared by immune cells developed from monocytes and macrophages and is possibly associated with an optimized search efficiency. To summarize, in this work, we employ the language of run-and-tumble to empower the research of microglial motility. The present framework can help guide future assessments of immune cell migration.

## Results

### Microglial kinematics and mechanotaxis

**Fig. 1.**
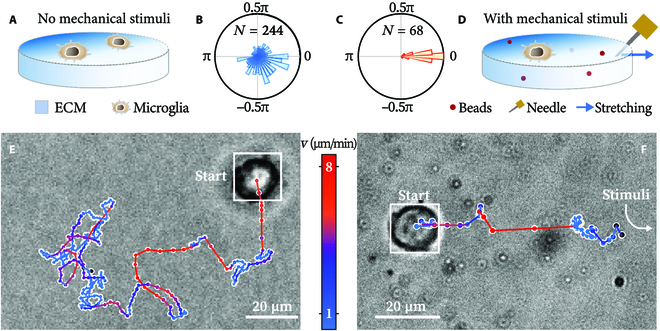
Microglia display clear mechanotaxis and run-and-tumble dynamics. Schemes of examining the microglial motion on ECM without (A) and with mechanical stimuli (D), and the respective distributions of the cells’ net moving directions (B and C). A typical cell moving without (E) and with (F) the presence of mechanostimuli. White boxes are the bounding boxes as a result of cell recognition, whose centers (white-edged circles) form the track. The track is colored by the cell’s instantaneous speed.

In vivo, microglia migrate in the extracellular matrix (ECM), which has intricate spatial organization and complex mechanical properties [17,20]. ECM serves as both the cells’ mechanical scaffold and the media where their biochemical and biomechanical dialogues take place [21]. To best simulate this microenvironment, we examine microglial migration on collagen, one of the primary components of ECM [20,22]. We track the single-cell locomotion of BV2 cells (a mouse microglial cell line) on ECM substrate, as schemed in Fig. 1A.

In the absence of external stimuli, the cells spend most of the time (84%) in an essentially quiescent state, during which they wobble locally. Nevertheless, intermittently, motility is activated. With customized software, we mark each cell’s track into the motile and quiescent states by thresholding their instantaneous velocity; see Supplementary Materials (SM) Section S1 and Figs. S1 and S2. During the motile state, the cells move at a 3- to 5-fold higher speed and move more persistently toward a direction. While transitioning between being motile or quiescent, the cells show no preferred moving direction (Fig. 1B) and their overall movement resembles a 2-dimensional random walk. A typical track is displayed in Fig. 1E (see also Movie S1).

On the contrary, when mechanical stimuli are present, microglial migration becomes highly directed (Fig. 1C, D, and F). The stimuli are induced by substrate stretching (Fig. 1D). A robotic arm holds a microneedle, inserts it into the ECM, and stretches the substrate. The arm is programmed to move in designed patterns. We implement cyclic stimuli consisting of an instant pull (0.3 s) and a wait of varying lengths (30 to 300 s); we track the cell motion responding to such stimuli. While still transitioning between the motile and quiescent states, all cells converge toward the stimuli; see Fig. 1C and F and Movie S2. The results prove that microglia are mechanotactic, which is previously unknown.

### Run-and-tumble dynamics of microglia

The pattern of microglia motility, both in explorative and tactic motion, is analogous to the renowned run-and-tumble motion of peritrichous bacteria. To explore this analogy, we benchmark the statistical features of microglial motion against those of bacterial run-and-tumble, which are summarized as follows [3,23]. (a) The motion consists of only 2 intertransitioning states, one responsible for net locomotion (run) and the other for turning (tumble); the intervals of both states follow exponential distributions. (b) The running direction is persistent but still subjected to rotational diffusion. (c) Tumbles reset the cell/microbes’ moving directions randomly. (d) The durations of consecutive runs and tumbles do not correlate. These features are covered sequentially in the following part.

#### Feature (a)

**Fig. 2.**
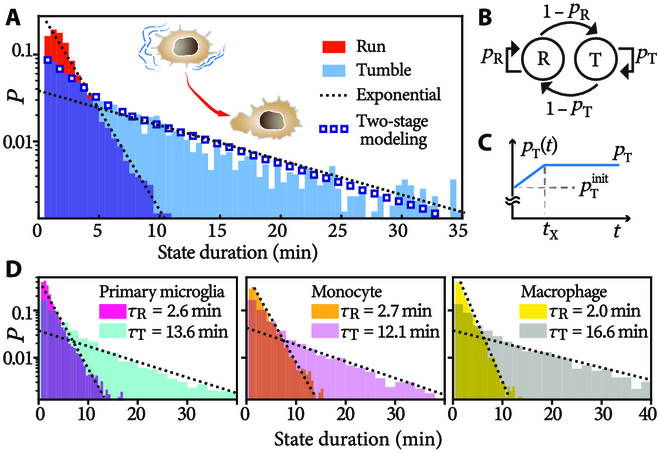
Modeling the state interval distributions. (A) Interval distributions of the motile (“run”) and quiescent (“tumble”) state of microglia (BV2 cells, *N* = 244) without stimuli. *τ_R_* = 1.8 min and *τ*_T_ = 10.9 min. Dotted lines: single-exponential fit. Blue squares: 2-stage modeling. (B) State machine representation of the run-and-tumble process. R and T stand for run and tumble, respectively. (C) *p*_T_ employed in the 2-stage model. (D) Same statistics in other immune cells.

We begin with examining the state interval statistics of microglial motility. Without external stimuli, the duration of single runs precisely follows the exponential distribution $P\left(t\right)=\frac{1}{\tau_{R}}e^{-t/\tau_{R}}$, where *τ*_R_ = 1.8 min, meaning that the probability for a run to continue after a unit time *t*_unit_ is $p_{R}=\frac{1}{t_{\text{unit}}}e^{-t_{\text{unit}}/\tau_{R}}=0.58\;\mathrm{mi}n^{-1}$ (Fig. 2A, data in red). For tumbles, except that the frequency for transient ones (<5 min) is underestimated, a single-mode exponential distribution already captures the results largely (*τ*_T_ = 10.9 min, *p*_T_ = 0.91 min^−1^, dotted line in Fig. 2A). An exponential distribution translates to a constant probability for a state to continue and therefore corresponds to a memoryless (Markovian) state evolution [3]. Noteworthy, the deviation from such a distribution in short tumbles points to a time-dependent *p*_T_, or in other words, a memory effect when the cells just start tumbling.

We thus capture the state interval distributions by Markovian and non-Markovian modeling respectively. Run-and-tumble process is represented as a state machine (Fig. 2B). The memory effect in tumbles is reproduced by a 2-stage model (Fig. 2C): Preceding a steady stage where *p*_T_ is a constant over time, there is an initial stage (*t* ≤ *t*_X_) where *p*_T_ begins with a smaller value, $p_{T}^{\text{init}}$, but later increases linearly to the steady-state constant. Quantitatively, $p_{T}^{\text{init}}$ is 0.83 min^−1^ and *p*_T_ saturates at 0.91 min^−1^ after *t*_X_ = 5 min; see the empty squares in Fig. 2A. *p*_T_ being initially lower means that, at the beginning of a tumble, cells have a stronger tendency to quit tumbling and become motile again.

In other macrophage-like cells, the same short-term memory effects are observed. Figure 2D presents interval statistics of mice primary microglia (*N* = 466, left panel), human monocyte (U937 cell line, *N* = 376, middle panel), and human macrophage (U937 cell line treated with phorbol 12-myristate 13-acetate, *N* = 143, right). Note that these cells all belong to the same immune cell subset that derived from monocyte, and their characteristic times are quantitatively similar to those of BV2 cells.

**Fig. 3.**
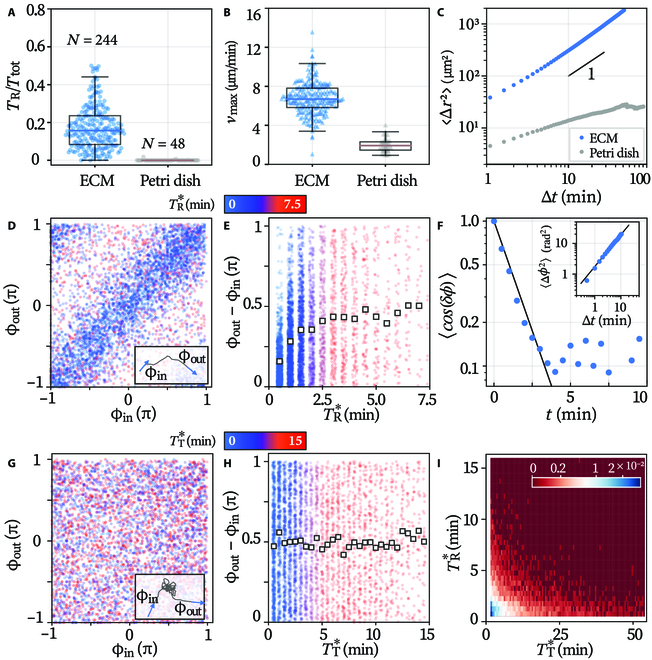
Microglial run-and-tumble dynamics. Time fraction of the motile state (A) and the cells’ maximum speed (B) on different substrates. (C) Mean square displacement over time. (D) Mapping between the *ϕ*_in_ and *ϕ*_out_ of runs. Inset: definition of *ϕ*_in, out_. (E) The net turning per run *ϕ*_out_ − *ϕ*_in_ as a function of run duration *T*_R_. Empty square: mean value over the binned events (colored points in the background). (F) Autocorrelation function of the running direction. Solid line: exponential fit. Inset: mean square angular displacement during the runs with fitting (solid line). (G) Correlation between *ϕ*_in_ and *ϕ*_out_ of tumbles. Inset: definition of *ϕ*_in, out_. (H) Net turning per tumble against tumbling interval *T*_T_. (I) Probability density of the duration of consecutive runs and tumbles. $T_{R,T}^{*}$ represent the state intervals with certain exclusions (Materials and Methods).

The run-and-tumble pattern gets activated only on certain substrates. On ECM substrates (2 to 200 Pa), microglia spend 16% of time running, with a maximum speed of 6.7 μm/min (median over the cells, Fig. 3A and B). The resultant migration is purely diffusive as the mean square displacement scales linearly with time (Fig. 3C). However, on uncoated polystyrene petri dish (∼GPa), microglia never become motile; see the much lowered motility metrics (Fig. 3A and B) and the plateaued mean square displacement curve at ∼40 μm ^2^ (i.e., displacement smaller than a cell size, Fig. 3C). More details can be found in Fig. S3.

#### Feature (b)

We next study the turning during runs. Figure 3D presents the relation between the incident (*ϕ*_in_) and outgoing angles (*ϕ*_out_) of runs. The angles are defined as shown in Fig. 3D inset. Each point represents a run event colored by its duration. The point cloud (N ≈5,000) distributes along the identity line (*ϕ*_in_ = *ϕ*_out_) with an overall correlation coefficient of 0.308 (Pearson’s *r*, same for the rest). Shorter runs (blue) are more concentrated along the identity line, indicating stronger correlation than the longer ones (red). This gradual loss of angular persistence over time is accentuated in Fig. 3E, where the average turning (〈*ϕ*_out_ − *ϕ*_in_〉) is plotted against the event duration. The turning becomes completely random after ∼2.5 min, as 〈*ϕ*_out_ − *ϕ*_in_〉 reaches *π*/2. Effect of the limited sampling rate is found to be insignificant; see Fig. S5 in SM Section S3.

This turning is effectively governed by rotational diffusion (Fig. 3F and inset). Diffusivity in angular space means that the mean square angular displacement (MSAD) of the moving direction *ϕ*(*t*) scales linearly with time as 〈Δ*ϕ*^2^〉 = 2*D_r_*Δ*t*. Δ*ϕ* = *ϕ*(*t*^′^ + Δ*t*) − *ϕ*(*t*^′^) is the turning in any interval of length Δ*t*. Meanwhile, the diffusivity also means that the autocorrelation function (ACF) of *ϕ*(*t*) decays exponentially over time as *e*^−*D_r_t*^ [24]. The ACF is computed as 〈**n**(0) · **n**(*δt*)〉 = 〈cos(*δϕ*)〉, with **n**(*t*) the unit vector along the moving direction and *δϕ* = *ϕ*(*t*) − *ϕ*(0) the net turning since *t* = 0. The exponential decay of ACF and linear scaling of MSAD over time are confirmed experimentally; see Fig. 3F. The diffusion coefficients *D_r_* obtained by fitting the ACF (0.70 min^−1^) and MSAD (0.64 min^−1^) agree well with each other. The corresponding time scale for rotational diffusion is *τ*_rot_ = 1/*D_r_* ≈ 1.5 min [24], which is comparably shorter than the duration of single runs (*τ*_R_ = 1.8 min). With *τ*_R_ being close to *τ*_rot_, runs of microglia may include obvious turnings, e.g., the middle fraction (red) of the track displayed in Fig. 1E.

Therefore, same as the runs of bacteria, those of microglia can also be considered as persistent motions subjected to rotational diffusion. While runs of bacteria are subject to hydrodynamic noise [3], those of microglia are probably dictated by noise of active origin [12]. While runs of bacteria are almost straight because of the event time being much smaller to the diffusive time scale (*τ*_R_ = 1 s < *τ*_rot_ ≈ 8 s [3]), runs of microglia are less persistent as these 2 time scales are comparable.

#### Features (c) and (d)

The turning by tumbles is presented in Fig. 3G and H in the same fashion as for runs. *ϕ*_in_ and *ϕ*_out_ of a tumble are respectively the cell’s direction at the end of the preceding run and that at the start of the following run (Fig. 3G inset). The uniformly distributed point cloud indicates no correlation between *ϕ*_in_ and *ϕ*_out_ (Pearson’s *r* = 0.000). Figure 3H further points out that the loss of angular persistence happens within a time scale smaller than our time resolution. Therefore, even a brief tumble completely resets a microglia cell’s moving direction.

Lastly, we confirm that the durations of consecutive states do not correlate. Run-tumble pairs formed by adjacent states (*N* ≈4000) are gathered and represented by their probability distribution on the *T*_T_ − *T*_R_ plane; see Fig. 3I. Same as bacterial run-and-tumble, the states’ duration is largely uncorrelated (correlation coefficient of −0.110).

Altogether, from the statistical point of view, the explorative migration of microglia on ECM is simply a different version of bacterial run-and-tumble. Nevertheless, besides exploring the ambient environment, another inherent goal encoded into bacterial run-and-tumble is to achieve targeted motion (chemotaxis) [4,25]. We move on to examine how tactic behaviors are realized by the run-and-tumble of microglia.

### Cell motility under inhibition of mechanosensing

While bacteria are spontaneously on the move by proton motive force [25,26], it appears that microglial motility needs to be activated by certain environmental cues. For example, microglia on petri dishes do not run (Fig. 3A to C). This points to a significant role of mechanosensing in activating microglial motility, and now, we clarify this role.

**Fig. 4.**
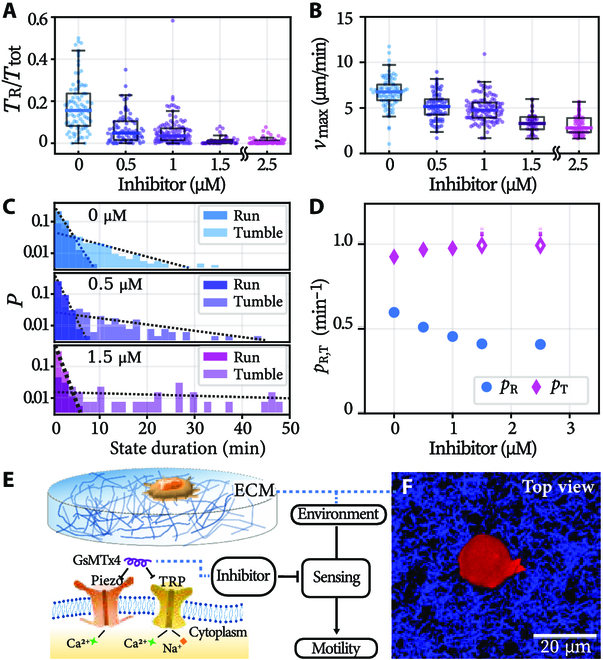
Motility modulation by mechanosensing. (A) Run-time fraction, (B) the maximum speed, and (C) representative interval distributions of cells subjected to increasing levels of inhibitor. Dotted lines: exponential fittings. (D) Probability for the run and tumble to continue, *p*_R, T_. Empty diamond with dashed line: estimated lower bound (Materials and Methods). (E) Schematics of microglia on ECM and the effect of GsMTx4. Flow chart: a minimal model for motility activation. (F) Confocal microscopy of a microglia cell (red) on ECM, with collagen fiber bundles in blue.

Experimentally, GsMTx4, a peptide that inhibits a cell’s mechanosensitive ion channels, is applied to cells at 0.5 to 3.0 μM concentrations. Both cells’ run-time fraction and the maximum speed plummet upon the use of inhibitor (Fig. 4A and B]. It is noteworthy that the 2 decreasing trends have slightly different meanings. While the decrease in run-time fraction (Fig. 4A) evidences the necessity of mechanosensing in motility activation, the decreasing maximum speed suggests that the cells’ sensing and motility are further coupled during runs.

We next resolve how mechanosensing modulates the run-and-tumble parameters. Representative state interval distributions are shown in Fig. 4C. The application of inhibitor appears to not affect the exponential nature. We extract the characteristic time of run and tumble, *τ*_R,T_, by fittings (dotted lines in Fig. 4C). The corresponding probabilities, $p_{R,T}=\frac{1}{t_{\text{unit}}}e^{-t_{\text{unit}}/\tau_{R,T}}$, are displayed in Fig. 4D. Additionally, cells treated with more than 2.5 μM inhibitor (*N* = 39) never run (*T*_tot_= 130 min of recording per cell, data not shown). This falling trend of *p*_R_ under increasing inhibitor concentration confirms clearly that the mechanosensing-related channels are required for motility activation; see Fig. 4E and F.

Recalling that motility emerges on ECM substrates but not on uncoated petri dishes (Fig. 3A to C), we further test whether this is because the cells have sensed a distinct substrate stiffness (∼GPa for petri dishes) from their native environments (10^0^ to 10^2^ Pa). We examine microglial run-and-tumble on petri dishes coated with poly (L-lysine)-graft-poly (ethylene glycol), a random graft co-polymer that renders a surface protein resistant [27]. As a result, microglia display the similar level of motility as they display on ECM substrates; see SM Section S2 and Fig. S4 for details. Altogether, the results suggest that both mechanosensing and chemical (protein) sensing are possibly involved in the activation of runs [12,28].

### Run-and-tumble migration in mechanotaxis

**Fig. 5.**
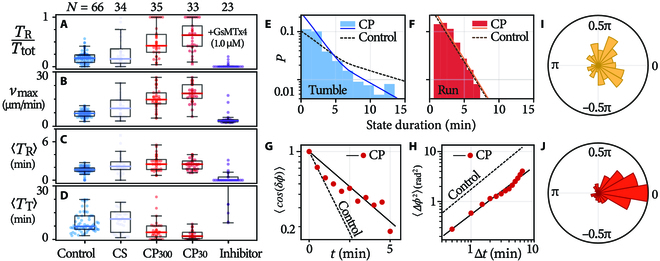
Microglial mechanotaxis. The run-time fraction (A), the maximum speed (B), and the mean duration of a single run (C) and tumble (D) of cells under different conditions. The interval distributions of tumbles (E) and runs (F) during mechanotaxis. (G) Autocorrelation function and (H) mean square angular displacement of runs during mechanotaxis. Dashed/solid lines in (E) to (H): best fits for the control group/CP groups. Directional distribution at the beginning (I) and at the end (J) of runs during mechanotaxis, computed by the first and the last 2 points of runs, respectively.

To achieve tactic behaviors (chemotaxis) is a goal inherently encoded into the run-and-tumble dynamics of bacteria [4,25]. Therefore, resolving how the run-and-tumble motion facilitates tactic behaviors will significantly deepen our understanding in this motion. We now resolve the strategies underlying microglial mechanotaxis and compare them with those adopted by bacteria to achieve chemotaxis. Microglial responses to different external stimuli are assessed. The test conditions include the following: stimuli-free (control), constant stretching, cyclic pulling of ∼300-s (CP _300_) and ∼30-s (CP _30_) periodicity (Materials and Methods), and under CP _30_ stretching but treated with 1 μM GsMTx4 (inhibitor). The motility of the different groups is shown in Fig. 5A to D. Inducing constant strain only enhances cell motility slightly (SM Section S4). However, when the strain rate increases (constant stretching, CP _300_, and the CP _30_ groups), motility is dramatically boosted: The run-time fraction is quadrupled (0.16, 0.42, and 0.64; Fig. 5A), and the maximum speed doubled (9.9, 14.8, and 18.1 μm/min; Fig. 5B). Finally, the mechanotactic motility is completely quenched by the inhibitor. With the highest strain rate (same as that of CP _30_), the inhibitor group stays largely, if not always, tumbling (Fig. 5A), which implies that the mechanical stimuli affected run-and-tumble motion via the responses of mechanosensitive ion channels.

We focus on the CP groups (including CP _30_ and CP _300_) and examine how the run-and-tumble statistics change during mechanotaxis. The interval distribution of runs remains identical to that of the control (Fig. 5F). Tumbles are still 2-staged but with sharply dropped persisting probabilities *p*_T_(*t*) (Fig. 5E). Runs during mechanotaxis become more persistent: The correlation coefficient between *ϕ*_in_ and *ϕ*_out_ increases to 0.379 (control: 0.308), and the diffusion constant decreases to *D_r_*= 0.30 (by ACF, control: 0.70; Fig. 5G) or 0.39 min^−1^ (by MSAD, control: 0.64; Fig. 5H). In mechanotaxis, tumble still randomizes a cell’s moving direction (correlation coefficient = 0.000). See SM Section S4 and Fig. S6 for more details.

Importantly, we find that microglia can steer promptly within a run. Figure 5I and J, respectively, display the cells’ moving direction when a run begins and ends. The directions begin dispersed, as they are randomized by the preceding tumble, and become focused toward the stimuli at the end (*ϕ* = 0, Fig. 5J). This ability to actively steer during taxis is a feat that most prokaryotes (e.g., bacteria) do not have [2].

The kinematic strategies underlying microglial mechanotaxis entail several aspects. First, microglia spend a larger fraction of time running: *T*_R_/*T*_tot_ is 4 times as large during mechanotaxis (Fig. 5A). Second, they run ∼3 times as fast (Fig. 5B). Last but most important is that they run more persistently, owing to their ability to steer. We highlight the last point by looking at the tumble frequency *f*_T_. As runs and tumbles always happen alternatively, *f*_T_ depends on the duration of single runs 〈*T*_R_〉 and tumbles 〈*T*_T_〉 as $f_{T}=\frac{1}{\langle T_{R}\rangle +\langle T_{T}\rangle }$. While 〈*T*_R_〉 is unchanged in mechanotaxis (Fig. 5C and F), 〈*T*_T_〉 plummets (Fig. 5D and E) and causes *f*_T_ to rise. This means that, in the targeted motion toward the stimuli, microglia lose track of the right direction more frequently. In this light, active steering (Fig. 5I and J) turns out to be the cornerstone of microglial mechanotaxis: Without it, faster and longer runs are futile for reaching the target as they will be along randomized directions.

## Discussion

In this work, we characterize microglial migration with and without the presence of external stimuli. As a result, we reveal a striking analogy between the microglial migration and the renowned bacterial run-and-tumble motion. We uncover qualitatively similar statistics in the state lifetime, turning behaviors, and state interval correlations of the two types of motion. We prove directly that microglia are mechanotactic, which is until now unconfirmed for this cell; we assess the cell’s strategies underlying mechanotaxis. While the biology behind bacterial run-and-tumble and chemotaxis are well resolved after decades of efforts [3,4,25], not much is known about the biology behind microglial mechanotaxis—except that mechanosensitive ion channels such as Piezo-1 are probably involved [29,30]. However, the elusive biology shall not hinder us from further analyzing the 2 types of motion and appreciating the analogy between such 2 distinct systems.

We also recapitulate the differences between the 2 versions of run-and-tumble, as they are the consequences of the 2 systems’ inherent distinctions. For example, bacteria live in a fluidic environment and they feed while swimming. The nutrient gradients in such an environment change dynamically because of diffusion [31,32], such that bacteria must keep sampling them. Therefore, bacteria have to be constantly on the move, and they must move fast enough to beat diffusion [33]. In contrast, microglia move to clear pathogens only when necessary, instead of to feed or to survive [14]. Also, in their environment, it is energetically more costly to move and diffusion is much less of a concern. Altogether, these help understand why bacteria spend 85% time running [4], whereas microglia only spend ∼16% (Fig. 3A), and why bacteria must run orders of magnitudes faster than microglia. The physical and biological distinctions between microglia and bacteria also manifest in their kinematic strategies. As eukaryotic cells, microglia have a more complex sensory and motility repertoire than bacteria. This is the backbone of their actively steered mechanotaxis (i.e., deterministic navigation [2]). On the contrary, bacteria cannot steer while running so they have to rely on stochastic turning, i.e., modulating the tumble frequency against the chemical gradient, to achieve chemotaxis [4,34,35].

Why do microglia run and tumble? For bacteria, the run-and-tumble motion is often analyzed and justified by their optimal feeding efficiency [5,32,36–38]. Following this paradigm, we examine whether microglial run-and-tumble is also optimal in fulfilling certain functions. We consider the patrol of microglia as a 2-dimensional diffusive searching process [11,39], and adopt the same notations as in [39] for consistency. During the search, a cell uses runs to cover a relatively large area of size *b*; it uses tumbles to inspect locally around itself, searching for particles of size *a*. Runs have a typical velocity of *V* while the tumbling search is described by the cell’s translational diffusion coefficient *D*. With this model, the optimal time for run (*τ*_R_) and tumble (*τ*_T_) to minimize the searching time depends on the aforementioned parameters (SM Section S5). Using realistic values of *b* = 100 μm, *a* = 0.2 μm, *V* = 5 μm/min, and *D* = 1 μm ^2^/min, we obtain $\tau_{R}^{\mathrm{opt}}=1.4$ min and $\tau_{T}^{\mathrm{opt}}=23.9$ min. More generally, varying (*a*, *b*, *V*, *D*) in the practical range gives $\tau_{R}^{\mathrm{opt}}∼O\left(10^{-1}-1\right)$, $\tau_{R}^{\mathrm{opt}}∼O\left(10-10^{2}\right)$, and resultant ratios of $T_{R}/T_{\mathrm{tot}}∼O\left(10^{-1}\right)$. The results are close to the observed values *τ*_R_ = 1.8 min and *τ*_T_ = 10.9 min in microglia (BV2 cells), as well as those for other immune cells, *τ*_R_ = 2 to 3 min and *τ*_T_ = 10 to 20 min (Fig. 2). Actually, the optimization of searching time may be the reason why these cells display extremely similar interval statistics [11]. In all, we present the possibility that microglial run-and-tumble represents a strategy that optimizes the searching efficiency.

Additionally, the question “why do microglia run and tumble” also leads to an intriguing possibility, that such motility is shared by a subset of immune cells. Microglia are resident macrophages in the brain [19]. In addition, macrophages in general, together with dendritic cells, develop from monocytes (a type of white blood cell) [40]. All these cells are found to display similar motility (Materials and Methods and [11]). Moreover, beyond this monocyte-derived subset, neutrophil, another type of white blood cell that differentiates from the same progenitor as monocyte, was reported to move in a reminiscent pattern [41]. To clarify exactly how similar/different the patterns of the aforementioned cells are, more research is called for and the run-and-tumble analogy provides a viable framework.

In this work, our primary focus is to employ the language of run-and-tumble, which is a decades-long paradigm in studying microbial motility, to describe and understand microglial kinematics. Just as the bacterial run-and-tumble is rarely discussed without chemotaxis, we resolve microglial motility in the context of mechanotaxis, which regulates microglial run and tumble states probably via the responses of mechanosensitive ion channels. Similarities, as well as distinctions, stand out from the analogy, and this helps better understand microglial motility. This study formulates a general framework for future assessments of immune cell searching strategies, as well as the motility of other tissue cells.

## Materials and Methods

### Cell culture

BV2 cells are obtained from the China Infrastructure of Cell Line Resource. Cells are cultured in Dulbecco’s modified Eagle medium (Gibco, C11995500BT) supplemented with 10% fetal bovine serum (Gibco, 10091-148) and 1% penicillin-streptomycin (Gibco, 15140-122). The cells are maintained under 5% CO_2_, 37 °C. When reaching up to 70% confluence, the cells are detached with 0.05% trypsin-EDTA (Gibco, 25300054) for 1 min and then resuspended in a fresh complete medium. The cell suspension (10^4^ ml^−1^) is added dropwise on petri dish (Falcon Easy-grip style, polystyrene, nonpyrogenic) or 1-mm-thick collagen gel (Type I collagen extracted from rat tail tendon, Corning, 354249) with normal osmolarity and a neutral pH. After the cells have landed on the substrate, they are observed in their maintaining condition with an inverted microscope (Nikon, Eclipse Ti).

### Setup

The patterns of microglial migration are examined on collagen substrates (2 to 6 mg/ml, ∼2 to 200 Pa). The working cell density corresponds to a mean distance between adjacent cells of ≈300 μm (Fig. S3).

Filamented borosilicate glass capillary tubes (Sutter, BF100-50-10) are pulled into needles with a tip diameter of ∼10 μm. Needles are held by a micromanipulator (Eppendorf, InjectMan 4) and inserted into the collagen gel to perform cyclic stretching. The needle is placed 100 μm to the right of the targeted cell (+*x*-axis). It enters 60 μm deep into the substrate and pulls the substrate 30 μm away from the cell. A stretch cycle consists of the following phases: pull (100 μm/s, <1 s), hold (30 s, 300 s, or ∞), release, and return (to the initial position, ∼6 s). For these assays, the collagen gel used is uniformly mixed with 0.013% w/v microbeads (0.87-μm diameter, Spherotech, FP-0856-2). The beads help track the gel deformation.

GsMTx4 TFA (MCE, HY-P1410A) is used as the inhibitor of mechanosensing. It inhibits cation-permeable mechanosensitive and strain-activated channels that belong to the Piezo and transient receptor potential channels families. The stock solution (1 mM GsMTx4 in Milli-Q water) is added to the suspension before cell deposition. In assays without mechanostimuli, cells are filmed every 0.5 min for 4 to 8 hours and tracked by template matching with customized software (OpenCV-python package [ver. 4.5.1]). In mechanotaxis assays, cells are filmed at the same rate but recordings last until they reach the needle tip region, and they are manually tracked. The cells’ net displacement with respect to the deforming gel substrate is obtained by subtracting the motion of the embedded microbeads. All tracks (*N* ≈10^3^) are checked by the user. Dirt and dead cells are discarded. As our focus is the single-cell kinematics, tracks wherein cells divide or collide (and stick to each other thereafter) are trimmed to 20 min before the event. We characterize a cell’s run speed primarily by its maximum value, *v*_max_. It is chosen over the mean or median speed for 2 reasons. First, the maximum speed of a cell is independent of state-marking process and is thus robust. Second, the maximum speed is more sensitive to changes in the running speed, as the erratic speed during tumble, which takes up 84% of the time per track, is not included.

### Data pooling

#### Cells under no external stimuli

The interval distributions Fig. 2A display *N* = 244 tracks on collagen substrates (2 to 6 mg/ml) and include *N* ≈5000 runs and tumbles. Events at the start and the end of each recording are not included. Extremely short events (*T*≤ 1 min) are not included. $T_{R,T}^{*}$ in Fig. 3 represents respectively the dataset after the mentioned exclusions. Additionally, $T_{R,T}^{*}$ in Fig. 3E and H are zeroed at 1 min for better visualization.

#### Cells subjected to inhibitor

Transient tumbles (*T*_T_ < 2.5 min) and those that last the entire recording are both not included for fitting (Fig. 4C). Under more than 1 μM inhibitor, the latter case becomes predominant, resulting in a large fraction of empty bins (Fig. 4C, bottom panel). Datasets with too few valid bin counts (<*N*_bins_/3) are excluded from fitting. Empirically, *N*_bins_= 50 for *T*_T_ ∈ [1, 50] min. If the fitted *τ*_T_ > 50 min, the lower bound 50 min is then reported (Fig. 4D).

#### Cells during mechanotaxis

*N* = 191 runs and *N* = 171 tumbles from *N* = 68 cells (CP groups) are pooled for the statistics shown. Runs and tumbles are assigned a weight of 1/*T*_tot_, with *T*_tot_ the video duration, to equalize the contribution per track. Because the most responsive cells may have one or no run-tumble switching during the recording, the beginning and ending intervals are included.

## Data Availability

The presented datasets are available upon request.

## Supplementary Materials

Supplementary 1 Sections S1 to S5. Figs. S1 to S7.
