# Supplementary material for: Run-and-Tumble Dynamics and Mechanotaxis Discovered in Microglial Migration
Source: Research (Wash D C). 2023 Mar 10:0063. doi: 10.34133/research.0063 (PMC10013966; doi:10.34133/research.0063)
Supplement: Supplementary 1 — Sections S1 to S5. Figs. S1 to S7. [file research.0063.f1.pdf]

Supplementary Materials for Run-and-tumble dynamics and mechanotaxis discovered in microglial migration

Yiyu Zhang[1,2†], Da Wei[1†], Xiaochen Wang[1,2,3], Boyi Wang[1,2], Ming Li[1,4], Haiping Fang[3,5], Yi Peng[1,2‡], Qihui Fan[1§], and Fangfu Ye[1,2,3,6*]

[1]Beijing National Laboratory for Condensed Matter Physics, Institute of Physics, Chinese Academy of Sciences, Beijing 100190, China
[2]School of Physical Sciences, University of Chinese Academy of Sciences, Beijing 100049, China
[3]Wenzhou Institute, University of Chinese Academy of Sciences, Wenzhou, Zhejiang 325000, China
[4]Songshan Lake Materials Laboratory, Dongguan, Guangdong 523808, China
[5]School of Science, East China University of Science and Technology, Shanghai 200237, China
[6]Oujiang Laboratory (Zhejiang Lab for Regenerative Medicine, Vision and Brain Health), Wenzhou, Zhejiang 325000, China
[*]Corresponding author. Email: fye@iphy.ac.cn
[§]Co-Corresponding author. Email: fanqh@iphy.ac.cn
[‡]Co-Corresponding author. Email: pengy@iphy.ac.cn
[†]These authors contributed equally to this work.

1 Marking motility states

Previously, the tumble in bacteria is marked by a large turning rate together with a drop in moving speed [1, 2]. The success of this algorithm depends on the tumbles being brief and the runs are mostly straight. However, neither of these applies to microglia. For microglia, the quiescent phase takes up the major fraction where there is no significant displacement to compute turning. Also, unlike bacteria, microglia may turn greatly during a single run [Fig. 1(c)]. Therefore, we mark the states primarily by the net displacement over a short time span.

The cell's maximum displacement ($\Delta r_{\mathrm{win}}^{\mathrm{max}}$) within the time window (t_{win}) helps mark the track

into inter-spaced sections of high (run) and low (tumble) motility. At each time instant, if the corresponding $\Delta r_{\text{win}}^{\text{max}} > R_{\text{thres}}$, it is marked as run and otherwise as tumble. Empirically, $t_{\text{win}} = 2\,\text{min}$ (N=5 frames) and $R_{\text{thres}} = 6\,\mu\text{m}$ for tracks longer than 12.5 min, which account for $\gtrsim 95\%$ of all the tracks; while $t_{\text{win}} = 1\,\text{min}$ (N=3 frames) and $R_{\text{thres}} = 5\,\mu\text{m}$ for the shorter tracks.

The two parameters, t_{win} and R_{thres} define the performance of state marker. t_{win} primarily determines the temporal resolution of state marking and should be as small as possible. R_{thres} primarily balances the fidelity of the marked states. For example, with a higher R_{thres}, runs are marked more strictly and thus have higher fidelity; however, meanwhile, the resultant tumbles become less accurate as they contain an increased fraction of runs.

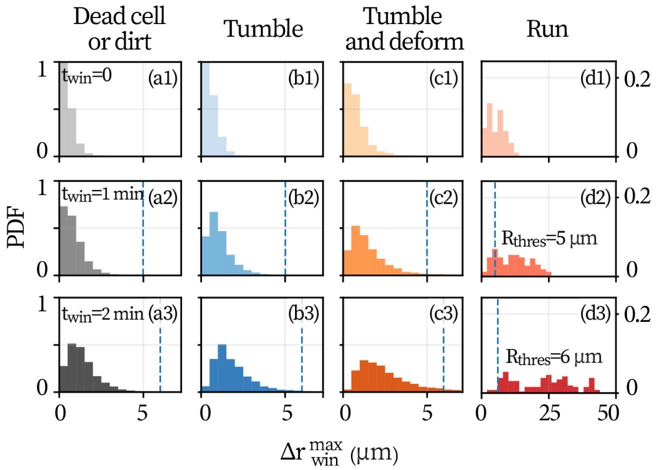

Figure S1: Distribution of $\Delta r_{\text{win}}^{\text{max}}$. Each column corresponds to results obtained from cells in a specific motility state; while each row corresponds to a specific setting of the state marker.

Parameter determination. The detected motion of a cell's center consists of three parts: the actual displacement, the displacement due to cell deformation, and tracking noise due to pixel value fluctuation. Parameters can be empirically determined by categorizing typical cell displacement in different states. Here we characterize typical displacement for runs, tumbles, deformation, and pixel fluctuation.

We prepare four training sets. The first one consists of dead cells or dirt that have similar sizes to microglia (N=19 in total, $\sim 80\,\text{h}$ of video) to benchmark the noise level of pixel fluctuation, see Figure S1(a1-3). The second one includes live cells that tumble throughout the recording without obvious shape change (N=21, $\sim 80\,\text{h}$); and this is to obtain the typical tumble motility, see Figure S1(b1-3). The third is comprised of tumbling cells that change shapes frequently (N=10, $\sim 20\,\text{h}$ of video). This set is used to evaluate displacement induced by cell deformation, see Figure S1(c1-3). The fourth dataset includes 85 min of recordings wherein the cells only run, see Figure S1(d1-3). In Figure S1, rows from top to bottom display distributions of the $\Delta r_{\text{win}}^{\text{max}}$ of frame-to-frame displacement ($N_{\text{win}} = 1$ frame), displacement over $t_{\text{win}} = 1\,\text{min}$ ($N_{\text{win}} = 3$ frames), and displacement over $t_{\text{win}} = 2\,\text{min}$ ($N_{\text{win}} = 5$ frames) respectively. The blue dashed lines mark the R_{thres} implemented in this study. Figure S1(b1) and (d1) highlight the necessity of not using the frame-to-frame displacement for state marking. The single-frame step sizes for the most motile cells (d1) overlap greatly

(∼ 20%) with the tumbling step sizes (b1). Meanwhile, pixel fluctuation (a1-3) and cell deformation (c1-3) do not significantly alter the detected tumbling motility (b1-3).

Lastly, we characterize the fraction of mis-marked states for the shown settings. For $(t_{\text{win}}, R_{\text{thres}}) = (1\,\text{min}, 5\mu\text{m})$, almost no tumbles will be mistaken as runs, as the total probabilities to the right of the dashed lines in Figure S1(b2) and (c2) are less than 1%. Meanwhile, ∼8% run will be marked as tumble. Note that this setting only applies short tracks ($T_{\text{tot}} \leq 12.5\,\text{min}$) which accounts for only 4% (N=15/369) of the cells used in the main text. For the majority (N=354/369) of the tracks, $(t_{\text{win}}, R_{\text{thres}}) = (3\,\text{min}, 5\mu\text{m})$. This leads to maximum 5% tumbles being mistaken as runs, see Figure S1(c3) and less than 1% runs being mistaken as tumbles (d3).

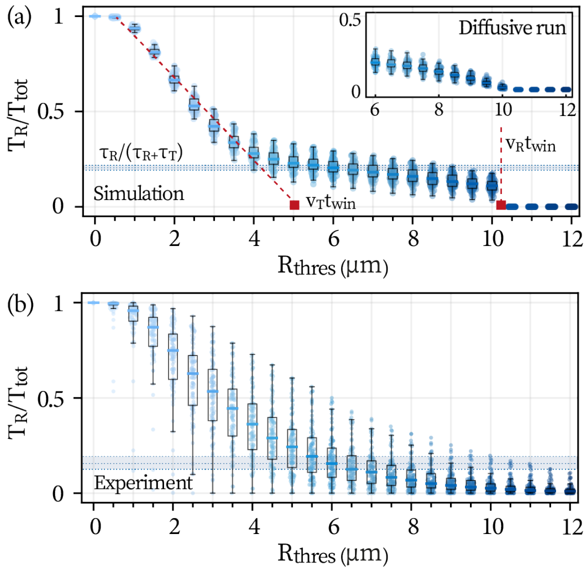

Figure S2: Determination of R_{thres}. The resultant run-time fractions $T_{\text{R}}/T_{\text{tot}}$ under varying R_{thres} are displayed, $t_{\text{win}} = 2\,\text{min}$. (a) Result with N=50 simulated tracks, where $v_{\text{R}} = 5.2\,\mu\text{m/s}$, $v_{\text{T}} = 2.6\,\mu\text{m/s}$, $\tau_{\text{R}}/\tau_{\text{T}} = 1/4$. Runs are diffusion free and tumble directions are completely random. Blue-shaded region: $T_{\text{R}}/T_{\text{tot}} = 0.2$. Inset: Adding rotational diffusion to runs. (b) N=89 tracks observed on 2 mg/ml collagen substrate without mechanostimuli. Blue-shaded range correspond to the median value of $T_{\text{R}}/T_{\text{tot}}(0.19\text{-}0.13)$ for $R_{\text{thres}} = 6 \pm 0.5\,\mu\text{m}$.

Understand the parameters. Here we show how R_{thres} and t_{win} are associated with the cells' motility. We simulate run-and-tumble tracks with known parameters. In the simulation, the particle (cell) switches between run and tumble at constant probability rates ($p_{\text{R,T}}$), giving rise to an exponential distribution of the state intervals. The characteristic time $\tau_{\text{R}}/\tau_{\text{T}}$ is set to 1:4. The particle runs at a constant speed v_{R} without turning. In tumbles, it changes direction randomly per time interval and moves one step forward at the speed of v_{R}. We apply the state marker to the simulated tracks and show how the marked run-time fraction $T_{\text{R}}/T_{\text{tot}}$ varies with R_{thres}, see Figure S2(a). The median value of the marked run-time fraction first drops quickly with R_{thres}. Extrapolating this drop (left red dash line), one obtains the tumble speed ($v_T t_{\text{win}}$) with the intercept (left red square). Further increasing R_{thres}, $T_{\text{R}}/T_{\text{tot}}$ decreases slowly around the set value $T_{\text{R}}/T_{\text{tot}} = \tau_{\text{R}}/(\tau_{\text{R}} + \tau_{\text{T}}) = 0.2$. Lastly, there is a cutoff at $v_R t_{\text{win}}$, as no particle will be able to move longer than that within t_{win}.

Adding rotational diffusion to the runs, the abrupt cutoff will be smeared, see the Figure S2(a) inset. To summarize, by setting the threshold at $R_{\text{thres}} \gtrsim v_{\text{R}} t_{\text{win}}$, one will be able to separate the runs from tumbles.

Therefore, we determine the R_{thres} for experimental data to be approximately 6 µm for $t_{\text{win}} = 2\,\text{min}$, see Figure S2(b). Due to the stronger noise in the experimental data, we examined the state marker working with R_{thres} ranging from 5 to 7 µm. No significant differences are found for the data presented here and for those in the main text.

2 Cell motility on different substrates

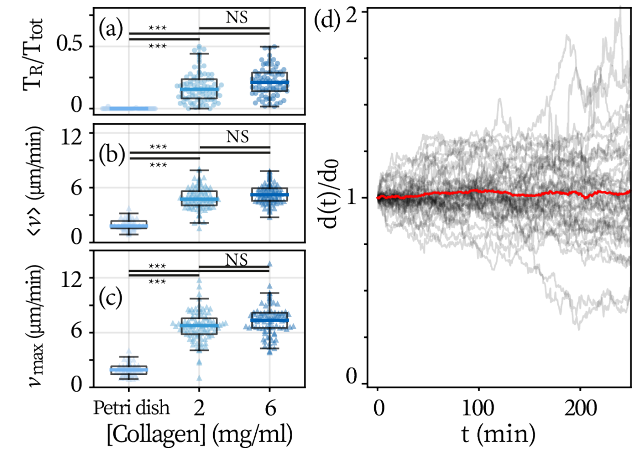

Figure S3: Microglial motility on different substrates. (a) Run-time fraction, (b) mean speed, (c) maximum speed of cells on Petri dish (∼GPa, N=48), 2 mg/ml collagen (∼2 Pa, N=89), and 6 mg/ml collagen (∼200 Pa, N=80). Significance is computed with Kruskal-Wallis test, a one-way ANOVA; NS: not significant, ***:P<0.001. (d) Cell-to-cell distance $d(t)$ on 2 mg/ml collagen substrate (N=70 pairs, initial distance d_0 <100 µm). Black lines: single pair data; red line: averaged over the shown population.

In Figure S3(a-c) we show the motility metrics of cells tested on different substrates. Cell groups on both the lowest (2 mg/ml) and the highest collagen concentrations (6 mg/ml) show significantly higher (P<0.001, Kruskal-Wallis test, a one-way ANOVA) motility than on Petri dish. However, groups on the collagen substrates show no significant difference between each other.

As moving cells may generate strains [3], it is possible that the baseline motility of microglia on ECM is activated by mechanotaxis towards the neighboring cell. Here we test and rule out this possibility. We gather N=70 pairs of cells on 2 mg/ml collagen substrate whose initial distances are smaller than 100 µm. The cell-to-cell distance over time $d(t)$ for each pair is normalized by their respective initial distance d_0, and displayed as a black trace in Figure S3(d). Overall, cells display no tendency to approach each other, as the average distance (red) shows no decreasing trend.

Microglial motility is further examined on Petri dishes treated with PLL(20k Da)-g-PEG(2k Da). The PEG side chains on a surface function as a water-binding hydrogel-like brush [4]. Adding PLL-

g-PEG slightly reduces cell motility for ECM substrates [P<0.05, KW test, Figure S4(a-b)], whereas it dramatically enhances all motility metrics for cells on Petri dishes (P<0.001, KW test, Figure S4). Hence, we conclude that the chemical properties of surfaces are also crucial for the activation of runs.

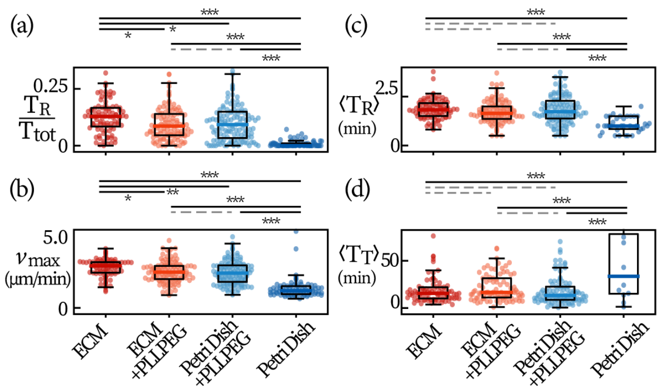

Figure S4: Microglial motility on functionalized hard surfaces. (a) Run-time fraction, (b) maximum speed, (c) mean run interval, and (d) mean tumble interval of BV2 cells on Petri dishes and ECM substrates. Significance is computed with Kruskal-Wallis test, a one-way ANOVA; *:P<0.05, **:P<0.01, ***:P<0.001, dashed line: not significant (P>0.05).

3 Effect of limited frame rate

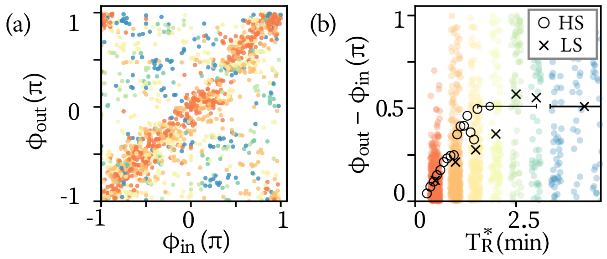

Figure S5: Effect of the limited frame rate on the loss of angular correlation during runs. (a) Angular correlation between ϕ_{in} and ϕ_{out} of runs in the LS dataset. (b) Effect of limited frame rate. HS: video taken at 5 s/frame; LS: HS down-sampled to 30 s/frame. Empty circles and crosses: mean of the binned events. Horizontal bar: range in T_R to compute the mean for the rightmost symbol. Colored dots are the LS results.

In the main text, the reported time scale for losing angular persistence during runs (τ_{rot}) is approximately 2 min [Figure 3]. This time scale is larger than yet comparable to our sampling time per frame (0.5 min). Therefore, here we quantify the effect of sampling rate on τ_{rot}.

Experiments with a 6× higher frame rate, fps=1/5 Hz are conducted. The dataset includes N=191 tracks, each lasting ∼1.5 h. These videos are down-sampled by a factor of 6, forming another dataset whose same sampling rate is the same as those shown in the main text. For simplicity, we refer to the two datasets as HS (high sampling rate) and LS (low sampling rate). Moving-window-

average of the track and the moving direction analysis are based on window size N=3 frames (1 min) and N=5 frames (20 s) for the LS and HS datasets respectively. The LS results [Figure S5(a-b)] are similar to their counterparts in the main text [Figure 3(d-e)]. Overlaying the HS result as open circles in Figure S5b, we see that the loss of angular persistence ($\langle \phi_{\mathrm{out}} - \phi_{\mathrm{in}} \rangle$ approaching $\pi/2$) happens at the same rate. Therefore, the effect of limited frame rate on the observed loss of angular persistence in runs is minor.

4 Motility in mechanotaxis

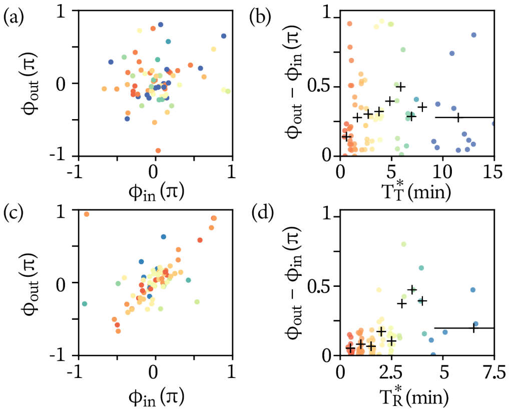

Figure S6: Correlation between ϕ_{in} and ϕ_{out} for tumbles (a-b) and runs (c-d) during mechanotaxis. Note that $\phi = 0$ is now the direction towards the mechanostimuli. Each point represents an event colored by its duration. Crosses: mean of binned events. Horizontal bar: range in $T_{\mathrm{R,T}}$ to compute the mean for the rightmost symbol.

Angular correlation in mechanotaxis. We confirm that microglial migration during mechanotaxis still bears the features of run-and-tumble motion. Figure S6(a)-(d) show similar mapping of the incident and outgoing angles (ϕ_{in} and ϕ_{out}) of runs and tumbles in mechanotaxis. N≈200 runs and tumbles in the CP groups (N=68 cells) are pooled. Panels are plotted in the same fashion as in Figure 3. Correlation coefficients between ϕ_{in} and ϕ_{out} are computed to be 0.379 for runs and 0.000 for tumbles (Pearson's r). In other words, angular persistence is lost gradually during a run and it is lost completely during a tumble. No qualitative difference from Figure 3 is observed.

Super-diffusive behavior. The 3D run-and-tumble motion of *E.coli* in chemotaxis is recently reported to be supper-diffusive with a MSD of $\langle \Delta r^2 \rangle \propto t^{1.66}$ [5]. We find a similar trend in microglia performing mechanotaxis. Figure S7(a) displays the MSD of the five groups of cells subjected to increasingly strong mechanostimuli. Scaling of the cells during mechanotaxis (the CP_{30} and CP_{300} groups) is $\langle \Delta r^2 \rangle \propto t^{1.6}$, similar to the bacterial motion in chemotaxis. While the latter is concluded to be a Lévy walk, our current data cannot prove whether microglial migration in mechanotaxis is of the same nature. The primary challenge here is the limited number of mechanotactic run events (∼200). To tackle this challenge, high-throughput mechanotaxis assays are called for, and we aim to resolve it in a future study.

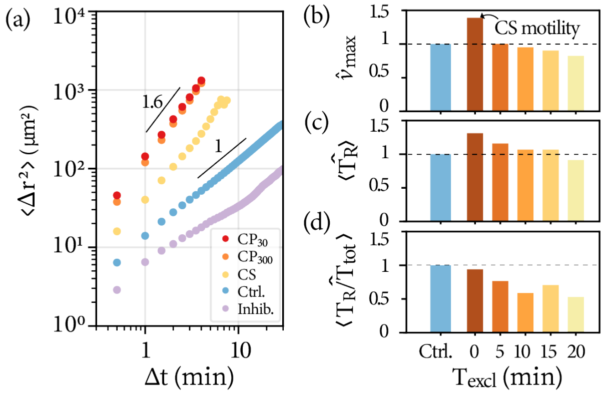

Figure S7: Mechanostimuli change microglial motion from diffusive to super-diffusive and temporal variation details of motility. (a) MSD of cells under different conditions. (b)-(d) Motility of the CS tracks after excluding the initial period $T_{\mathrm{excl.}}$ of each track. $\hat{}$: metrics normalized by their counterparts of the control group. CP: cyclic pulling; CS: constant strain.

Motility variation over time. The pull-hold-and-release scheme employed in this study will induce mechanostimuli that varies over time. We explore this temporal motility variation by examining tracks of the constant strain (CS) group. We cut off the initial period of each track ($T_{\mathrm{excl.}}$), and study the motility of with the trimmed tracks. Figure S7(b-d) compares the maximum speed (b), the average duration of a single run (c), and the average run-time fraction (d), of the trimmed tracks. The y-axes are normalized by their counterparts in the control group. Clearly, the observed motility enhancement in the CS group ($T_{\mathrm{excl.}} = 0$) disappears - if the initial 5 min is excluded. In fact, all motility metrics have dropped to either a comparable or even a lower level than the control group after the initial 5 min (four leftmost bars). One possibility underlying this effect is that the cells sensed the strain rate, and the strain rate becomes negligible as the strain saturates after $\sim 5\,\mathrm{min}$.

5 The optimal time for run and tumble

In Ref.[6], the authors provide an analytical expression for the optimal time for run (τ_{R}) and tumble (τ_{T}) to minimize the first-passage time in a 2D diffusive searching process:

$$\tau_{\mathrm{R}}^{\mathrm{opt}} \approx \frac{b^2}{D}\frac{\mathcal{C}}{w^2(\mathcal{C}-2)}, \tau_{\mathrm{T}}^{\mathrm{opt}} \approx \frac{b}{V}\frac{\mathcal{C}^{-\frac{1}{2}}}{w}. \tag{1}$$

The coefficients $\mathcal{C} = 4\ln w - 5 + c$ and w are obtained by solving a equation solely dependent on (a, b, V, D):

$$\begin{aligned}\frac{2Vb}{wD}\ln\mathcal{C} = &-8\ln^2 w + (6 + 8\ln\frac{b}{a})\ln w \\ &- c(\frac{c}{2} + 2\ln\frac{b}{a} - \frac{3}{2}) + 11.\end{aligned} \tag{2}$$

Here $c = 4(\gamma - \ln 2) \approx -0.4637$ with γ the Euler constant.

References

[1] H. C. Berg and D. A. Brown, "Chemotaxis in escherichia coli analysed by three-dimensional tracking," *Nature*, vol. 239, no. 5374, pp. 500–504, Oct. 1972, ISSN: 1476-4687. DOI: `10.1038/239500a0`.

[2] U. Alon *et al.*, "Response regulator output in bacterial chemotaxis," *EMBO J.*, vol. 17, no. 15, pp. 4238–4248, 1998.

[3] Q. Fan *et al.*, "Dynamically re-organized collagen fiber bundles transmit mechanical signals and induce strongly correlated cell migration and self-organization," *Angew. Chem.*, vol. 60, no. 21, pp. 11 858–11 867, 2021.

[4] R. Marie, J. P. Beech, J. Vörös, J. O. Tegenfeldt, and F. Höök, "Use of pll-g-peg in microfluidic devices for localizing selective and specific protein binding," *Langmuir*, vol. 22, no. 24, pp. 10 103–10 108, 2006, PMID: 17107006. DOI: `10.1021/la060198m`. eprint: `https://doi.org/10.1021/la060198m`. [Online]. Available: `https://doi.org/10.1021/la060198m`.

[5] H. Huo, R. He, R. Zhang, and J. Yuan, "Swimming escherichia coli cells explore the environment by lévy walk," *Appl. Environ. Microbiol.*, vol. 87, no. 6, G. Alexandre, Ed., e02429–20, 2021. DOI: `10.1128/AEM.02429-20`.

[6] O. Bénichou, C. Loverdo, M. Moreau, and R. Voituriez, "Intermittent search strategies," *Rev. Mod. Phys.*, vol. 83, pp. 81–129, 1 Mar. 2011.
